# Dual‐Functional High‐Entropy Polymer Exhibiting Giant Cross‐Energy Couplings at Low Fields

**DOI:** 10.1002/smsc.202400624

**Published:** 2025-02-22

**Authors:** Guanchun Rui, Wenyi Zhu, Li Li, Jongcheol Lee, Yiwen Guo, Qin Zou, Siyu Wu, Ruipeng Li, Thierry Lannuzel, Fabrice Domingues Dos Santos, Mark A. Aubart, Seong H. Kim, Long‐Qing Chen, Lei Zhu, Zi‐Kui Liu, Q. M. Zhang

**Affiliations:** ^1^ Arkema Inc. 900 First Avenue King of Prussia PA 19406 USA; ^2^ School of Electrical Engineering and Computer Science Materials Research Institute The Pennsylvania State University University Park PA 16802 USA; ^3^ Department of Materials Science and Engineering The Pennsylvania State University University Park PA 16802 USA; ^4^ Department of Chemical Engineering The Pennsylvania State University University Park PA 16802 USA; ^5^ Department of Macromolecular Science and Engineering Case Western Reserve University Cleveland OH 44106 USA; ^6^ National Synchrotron Light Source II Brookhaven National Laboratory Upton NY 11973 USA; ^7^ Arkema‐Piezotech Rue Henri‐Moissan 69493 Pierre‐Benite Cedex France

**Keywords:** electrocaloric, electrostriction, ferroelectric polymers

## Abstract

A key component of cooling devices is the transfer of entropy from the cold load to heat sink. An electrocaloric (EC) polymer capable of generating both large electrocaloric effect (ECE) and substantial electroactuation can enable EC cooling devices to pump heat without external mechanisms, resulting in compact designs and enhanced efficiency. However, achieving both high ECE and significant electroactuation remains challenging. Herein, it is demonstrated that poly(vinylidene fluoride‐trifluoroethylene‐chlorofluoroethylene‐double bond) [P(VDF‐TrFE‐CFE‐DB)] tetrapolymers can simultaneously generate high electrocaloric effects and electroactuations under low fields. These P(VDF‐TrFE‐CFE‐DB) tetrapolymers are synthesized through the dehydrochlorination of P(VDF‐TrFE‐CFE) terpolymer. By facile tuning the composition of the initial terpolymer to avoid pure relaxor state, tetrapolymers with optimal DB compositions are achieved, near the critical endpoint of normal ferroelectric phase with diffused phase transition. The nearly vanishing energy barriers between the nonpolar to polar phases result in a strong electrocaloric response and significant electroactuation. Specifically, the P(VDF‐TrFE‐CFE‐DB) tetrapolymer exhibits an EC entropy change Δ*S* of 100 J kg^−1^ K^−1^ under 100 MV m^−1^: comparable to state‐of‐the‐art (SOA) EC polymers, while delivering nearly twice the electroactuation of the SOA EC polymers. This work presents a general strategy for developing EC materials that combine large electrocaloric effect and electroactuation at low electric fields.

## Graphical abstract

Multifunctional ferroelectric polymer exhibits simultaneously giant electrocaloric entropy changes (100 J kg^−1^ K^−1^) and electroactuation (4.3%) effects. By introducing double bonds to tune the compositions near a critical endpoint of the normal ferroelectric phase, the energy barriers between the nonpolar and polar phases nearly vanish. This founding promotes the development of self‐actuated EC devices.

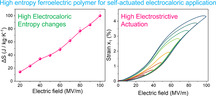

## 1 Introduction

Modern cooling technologies are facing significant environmental and energy challenges. Refrigerants in modern cooling vapor‐compression cooling, (VCC) are a major cause of greenhouse gas emission.^[^ ^1^ ^]^ Cooling and refrigeration overall consume 30% of electricity in the USA and about 20% worldwide.^[^ ^2^, ^3^ ^]^ All these call for developing alternative cooling technologies that are environmentally benign and exhibit higher energy efficiency than VCC.

Applying an electric field to a dielectric material will induce a change in the polarization and, consequently, a change in the entropy and temperature in the material. Such an electric field‐induced reversible entropy and/or temperature change in a dielectric is known as the electrocaloric effect (ECE).^[^ ^2^, ^3^, ^4^, ^5^ ^]^ Thus, EC working body is essentially a dielectric capacitor. Charging and discharging a dielectric capacitor causes very little energy loss. Hence, EC cooling is greenhouse gas‐free and has the potential to achieve higher efficiency than VCC‐based cooling. The large electrocaloric effect near room temperature discovered in ferroelectric polymers and ceramics^[^ ^5^, ^6^, ^7^, ^8^, ^9^ ^]^ has enabled the demonstration of EC cooling devices and modules with attractive and promising performance.^[^ ^10^, ^11^, ^12^, ^13^, ^14^, ^15^, ^16^, ^17^, ^18^ ^]^

One key component of a cooling device is the transportation of entropy (heat) from one temperature level to another temperature level. In EC cooling devices, such process is often achieved by external means, such as pumps, motors, or electrostatic force, which consumes additional energy.^[^ ^10^, ^11^, ^12^, ^13^, ^14^, ^15^, ^16^, ^17^ ^]^ Ferroelectrics are multifunctional, for example, a ferroelectric material may exhibit both large electroactuation in addition to a large ECE. Such an EC material could enable EC cooling devices to pump heat between two ends without external mechanisms, thus reducing device size and improving efficiency.^[^ ^18^, ^19^, ^20^ ^]^ Very recently, Han et al. reported a self‐oscillating polymer refrigerator which makes use of both large ECE and electromechanical actuation, thus removing the need for extra‐deriving system to pump heat between the cold and hot ends, leading to a high efficiency.^[^ ^20^ ^]^

In the self‐oscillating polymer refrigerator, the EC tetrapolymer that generates large ECE at ultralow electric fields reported in ref. [9] was used as the starting EC material. However, the actuation strain, that is, 1%, was too small to move the EC polymer device to generate a gap with a meaningful temperature span of *T*_h_–*T*_c_.^[^ ^20^ ^]^ To reach a higher actuation strain, Han et al. traded the EC performance of the P(VDF‐TrFE‐CFE‐DB) tetrapolymer with the electroactuation. It was shown that increasing the DB content in the tetrapolymer from 0.6 to 0.8 mol%, which reduced ECE slightly, led to a relatively larger electroactuation (≈1.9% under 70 MV m^−1^ electric field) and demonstrated the concept.^[^ ^20^ ^]^

For self‐pumping polymer refrigerators, a large electroactuation strain at low electric fields is desired. We investigate a novel approach for EC polymers to generate both large electroactuation and large ECE: The dual functional electrocaloric polymer exhibits giant ECE and electroactuation concomitantly, that is, giant cross‐energy couplings at low fields.

Earlier studies showed that introducing proper amount of double bonds or functionalization in P(VDF‐TrFE)‐based polymers can greatly impact the ferroelectric performance of the polymers.^[^ ^9^, ^21^, ^22^ ^]^ In, ref. [9], X. Qian et al. showed that converting CFE in the P(VDF‐TrFE‐CFE) 65/35/7 mol% (we label the composition as the VDF/TrFE ratio, CFE mol%, and DB mol% in the terpolymers and tetrapolymers) relaxor polymer into double bond (DB) weakened the relaxor response of the polymer, and at a critical DB content, the tetrapolymer exhibited a high electrocaloric response at low electric field. For the tetrapolymers in refs. [9, 20], this critical DB composition is 0.6 mol% of DB content in the tetrapolymer. Further increasing the DB content resulted in a reduction of electrocaloric effect. In the tetrapolymer here, the defect monomer CFE has a larger size while DB monomer unit has a smaller size compared with VDF and TrFE (see Figure S3, Supporting Information) and, when used together, is effective in tuning the phase transition behaviors of P(VDF‐TrFE) polymers.^[^ ^23^, ^24^ ^]^ Including the bulky CFE in P(VDF‐TrFE) weakens the ferroelectric response and P(VDF‐TrFE‐CFE) terpolymer with 7 mol% CFE exhibits characteristics of pure relaxor. On the other hand, reducing the bulky CFE content in the terpolymer weakens the relaxor response of the polymer.^[^ ^23^, ^25^ ^]^ In the terpolymer studied in refs. [9, 20], the CFE content is 7 mol%. We hypothesize that if the initial terpolymers contain a lower CFE content than those in refs. [9, 20], the resulting tetrapolymers may generate both high ECE and electroactuation at an optimal DB content.

Here, we synthesized P(VDF‐TrFE‐CFE) 67/33/6.6 terpolymer which has a lower CFE content. The terpolymers were converted to P(VDF‐TrFE‐CFE‐DB) 67/33/6.6‐x/x tetrapolymers by de‐hydrochlorination (de‐HCl) method.^[^ ^9^, ^21^, ^22^ ^]^ We show that the tetrapolymer 67/33/4.6/2 mol% exhibits both high ECE and electroactuation. We further show that the 67/33/4.6/2 mol% tetrapolymer has been converted to a normal ferroelectric near the critical point with a diffused phase transition. It is the nearly zero energy barrier between the nonpolar and the polar phases in the broad critical region of this tetrapolymer that enable large ECE and large electroactuation under low electric fields.

## 2 Results and Discussion

### 2.1 Cross‐Energy Couplings for the PVDF Tetrapolymers

**Figure 1:**
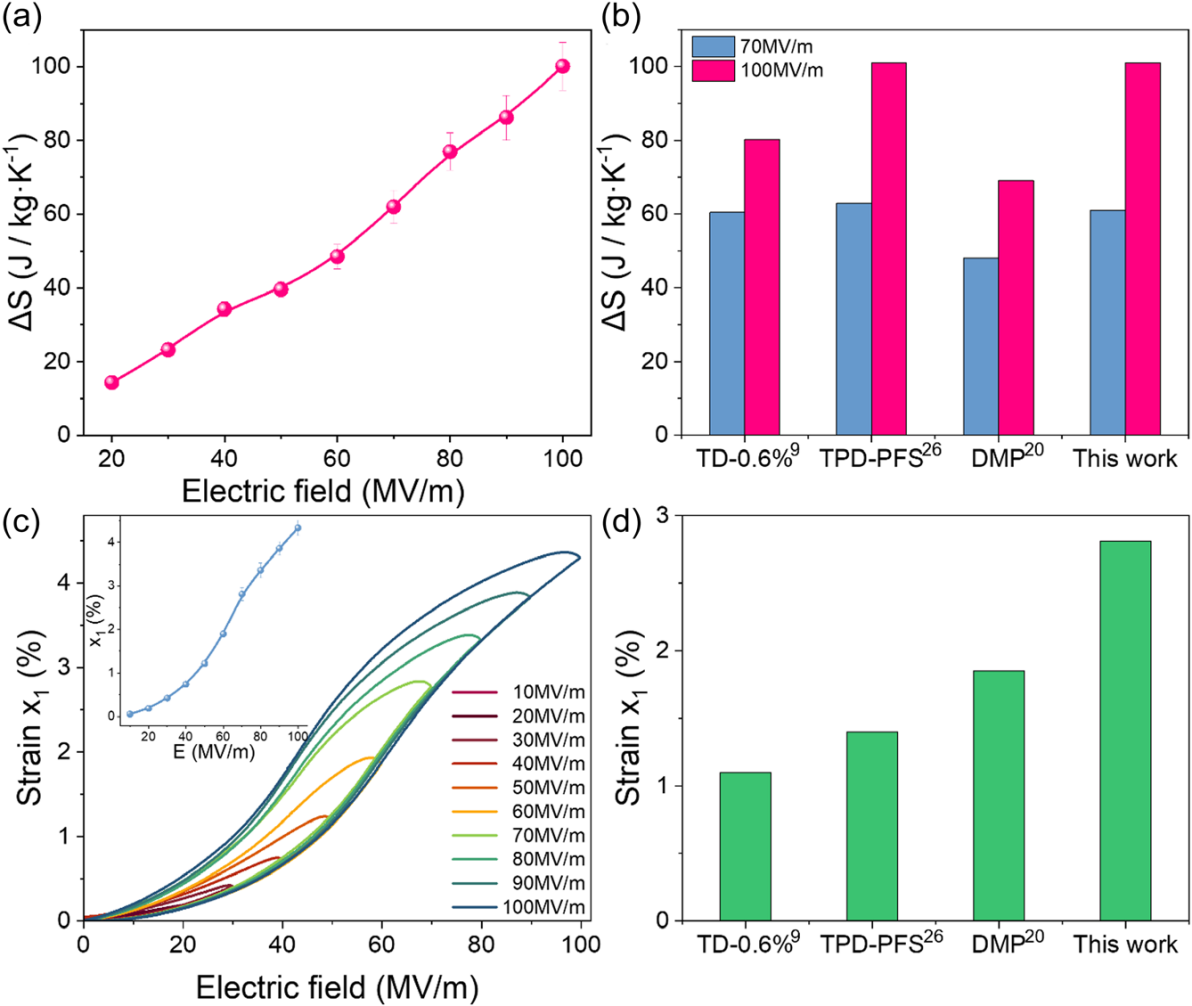
a) EC Δ*S* versus the applied fields for the tetrapolymer 67/33/4.6/2 mol% and b) comparison of the EC Δ*S* with the state‐of‐the‐art EC polymers in the literature. c) Electroactuation strains *x*_1_ of the tetrapolymer and d) comparison of *x*_1_ with the state‐of‐the‐art EC polymers (at 70 MV m^−1^). The error bar in (a,c) showing the mean value ± SD, *n* = 6.

We characterized ECE and electrostrictive strain *x*_1_ of the P(VDF‐TrFE‐CFE) 67/33/6.6 mol% terpolymer and the tetrapolymers derived from it. We observed that the tetrapolymer 67/33/6.6‐n/n with 2.0 mol% DB (*n* = 2.0, see the NMR result in Figure S4, Supporting Information) exhibited the largest ECE (Figure S5, Supporting Information) and largest electroactuation *x*_1_ among the tetrapolymers synthesized. In the EC refrigerators, it is the *x*_1_, the electroaction strain along the film surface, that “pumps” the heat of EC polymers from the cold end to a heat sink.^[^ ^19^, ^20^ ^]^ As shown in Figure S6, Supporting Information, the tetrapolymer 67/33/4.6/2% exhibits a high ECE and a high electroactuation *x*_1_ in the temperature ranges studied (297 to 333 K). The EC Δ*S* reaches 100 J kg^−1^ K^−1^ under 100 MV m^−1^ at 323 K, as presented in **Figure** **1**a, which is on a par with the state‐of‐the‐art EC polymers,^[^ ^26^ ^]^ Figure 1b, and higher than that reported in refs. [9, 20]. Figure 1c is *x*_1_ of the tetrapolymer with 2 mol% of DB measured at the same temperature (323 K), which can reach 2.75% under 70 MV m^−1^, higher than 1.9% under the same field of the DB tetrapolymers^[^ ^20^ ^]^ (see Figure 1d), as well as other EC polymers exhibiting large ECE.^[^ ^9^, ^26^ ^]^ The results presented demonstrate that in optimally designed DB tetrapolymers, both large ECE and large electroacuation under low electric fields can be achieved concomitantly.

### 2.2 Dielectric Behaviors of the PVDF Tetrapolymers

**Figure 2:**
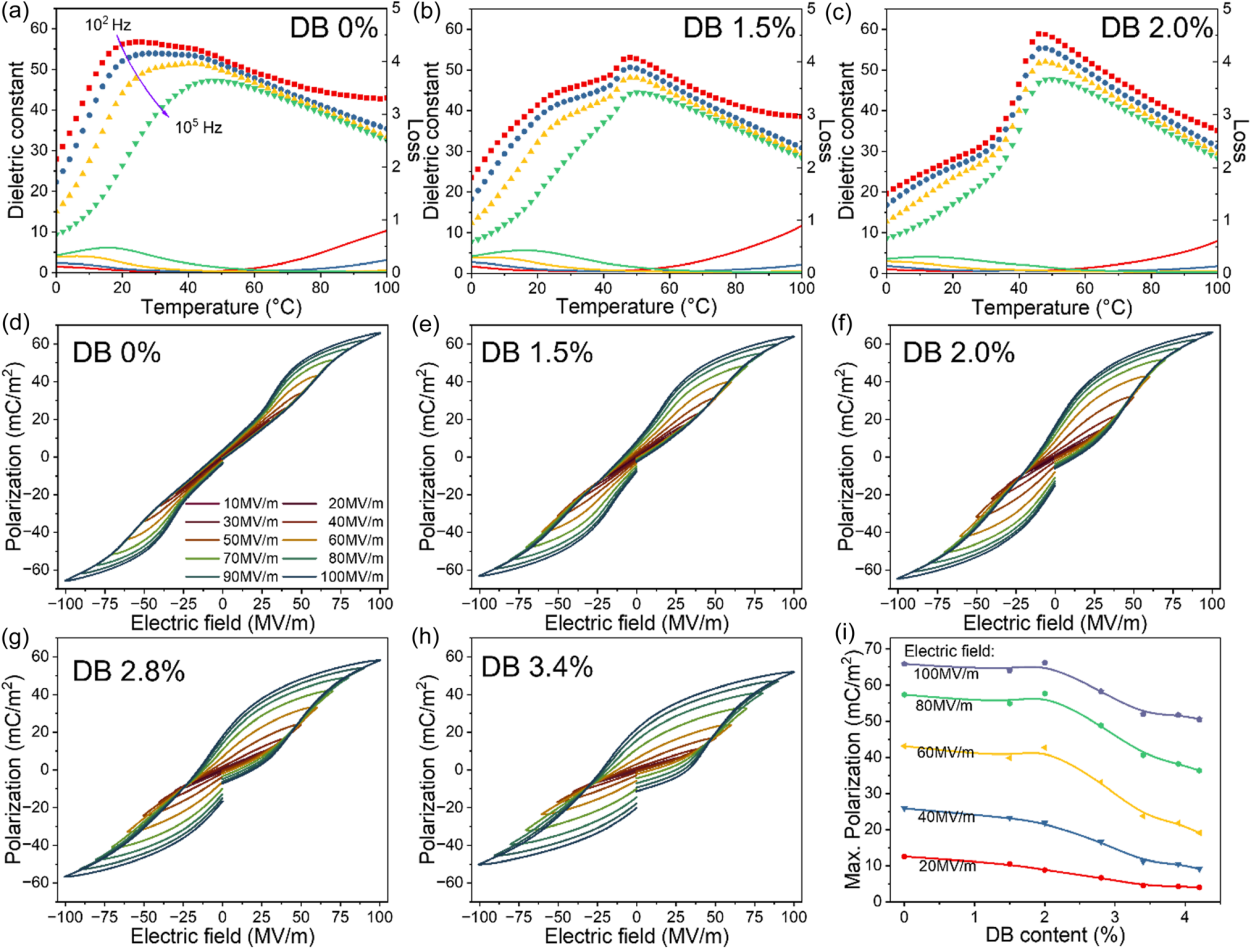
The dielectric properties versus temperatures of a) the initial terpolymer and b,c) the tetrapolymers at 1.5 and 2 mol% of DB content. The polarization‐electric field (*P*–*E*) bipolar loops for d) the initial terpolymer and e–h) tetrapolymers with different DB contents under different electric fields. i) The peak polarization of the *P*–*E* loops at different applied electric fields versus DB contents.

To provide an understanding of the giant ECE and electroactuation of the tetrapolymer P(VDF‐TrFE‐CFE‐DB) 67/33/4.6/2, we characterized the dielectric properties of the tetrapolymers. Presented in **Figure** **2**a are the dielectric properties of P(VDF‐TrFE‐CFE) 67/33/6.6 at different frequencies as functions of temperature. The data reveals that in addition to the dielectric behavior characteristic of relaxor ferroelectric, that is, a broad dielectric peak which shifts to higher temperature with frequency, there is a weak shoulder at temperatures at about 40 °C, indicating that the terpolymer is not in pure relaxor phase. This is distinctively different from the terpolymers in refs. [9, 20] (65/35/7 mol%) (Figure S7b, Supporting Information), as well as the terpolymer 68/32/7.3 mol% (Figure S7a, Supporting Information), reported in refs. [24, 27] (see Figure S8, Supporting Information).

Presented in Figure 2b,c are the dielectric properties of the tetrapolymers with 1.5 and 2.0 mol% of DB contents, which illustrate the growth of a dielectric peak at ≈46 °C, and for the tetrapolymer with 2 mol% DB, the relaxor peaks disappeared and the dielectric response is dominated by a broad peak centered at ≈46 °C whose position does not change with frequency. Figure S9, Supporting Information, presents the dielectric properties of more tetrapolymer compositions. The evolution of the dielectric properties with DB content of the tertrapolymers, derived from the terpolymer (67/33/6.6), is very different from those reported earlier, which were derived from the terpolymers at compositions of a pure ferroelectric relaxor.^[^ ^9^, ^20^, ^24^, ^27^ ^]^ Here, the tetrapolymer with 2 mol% DB exhibits a diffused phase transition between the low‐temperature phase and a high‐temperature nonpolar phase. In contrast, the tetrapolymer generates the highest ECE in ref. [9] while a low electroactuation still exhibits a strong relaxor dielectric behavior (see Figure S10, Supporting Information). The electric field‐induced phase transition in the diffused transition region generates large ECE and electroactuation at low electric fields.

We characterized the bipolar polarization‐electric field (*P*–*E*) loops for the 67/33/6.6 P(VDF‐TrFE‐CFE) terpolymer and the DB tetrapolymers derived from it at 10 Hz applied fields, which are presented in Figure 2d–h. The data shows that the peak polarization from the bipolar *P*–*E* loops does not change with the DB content till the tetrapolymer with 2 mol% DB content. Beyond that, the polarization P decreases with the DB content in the tetrapolymers. These results suggest the tetrapolymer at 2 mol% composition is at a transition point of the ferroelectric response, consistent with the dielectric data in Figure 2c and Figure S9, Supporting Information. At higher DB contents (2.8 to 4.2 mol%), the *P*–*E* loops are dominated by the normal ferroelectric behavior. Increased coercive fields cause a reduced peak polarization at electric fields below 100 MV m^−1^ for these tetrapolymers.

It is common for ferroelectric systems to adopt a metastable equilibrium state near the field‐induced phase transitions. In this regime, energy barriers are minimized and even a small change in the applied field can lead to a large change in dielectric and other properties.^[^ ^28^, ^29^, ^30^, ^31^ ^]^ For instance, near the Curie transition temperature θ, the free energy of the polar and the nonpolar phases become nearly the same, leading to the so‐called Curie–Weiss law *K* = *C*/(*T*‐*θ*), where *K* is dielectric constant (or dielectric permittivity *ε* = *K*ε_0_ where *ε*_0_ = 8.85 × 10^−12^ F/m is the vacuum permittivity).^[^ ^28^ ^]^ At the transition region, other functional properties such as EC Δ*S*/Δ*E* and electromechanical Δ*x*/Δ*E* also exhibit the highest values.^[^ ^29^, ^30^, ^31^ ^]^ Broadening the phase transition region by employing various approaches to induce diffused phase transition will lead to large K, Δ*S*/Δ*E*, and Δ*x*/Δ*E* over a broader temperature range. The results here are consistent with these considerations. On the other hand, we feel like these observations may be distinct from the morphotropic phase boundary theory, which usually describes the coexistence of two conformations in ferroelectrics.^[^ ^21^, ^32^, ^33^ ^]^ During the diffuse transition, multitypes of conformational changes could happen at low fields and thus endow larger ECE entropy changes.^[^ ^30^ ^]^

### 2.3 Structural Bias for Cross‐Energy Coupling inside the PVDF‐Based Tetrapolymers

**Figure 3:**
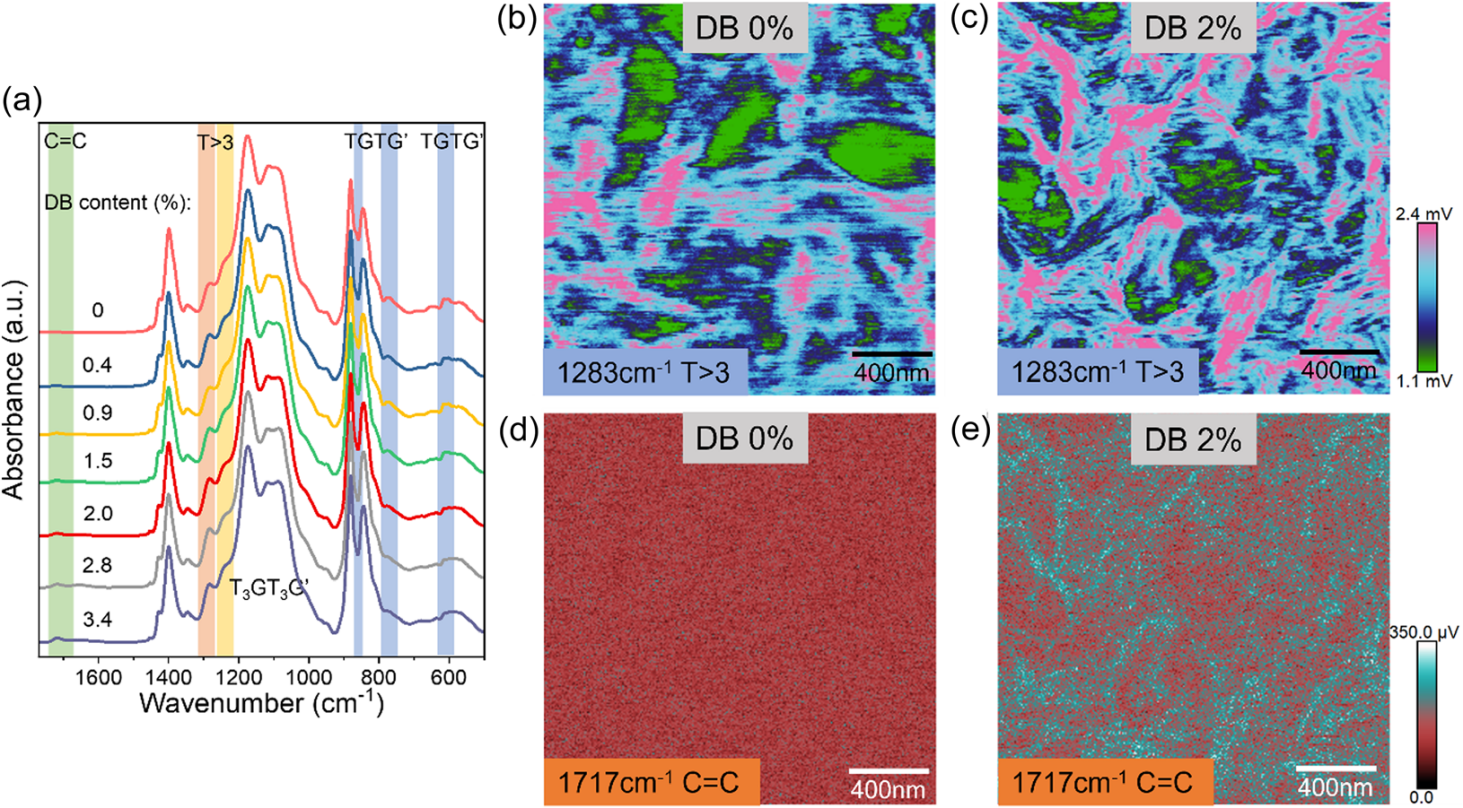
a) FTIR spectra of the tetrapolymers with different DB contents. AFM‐IR mapping for b,d) DB 0% terpolymer and c,e) DB 2% tetrapolymer at the scanning frequency of b,c) 1283 cm^−1^ and d,e) 1717 cm^−1^.

**Figure 4:**
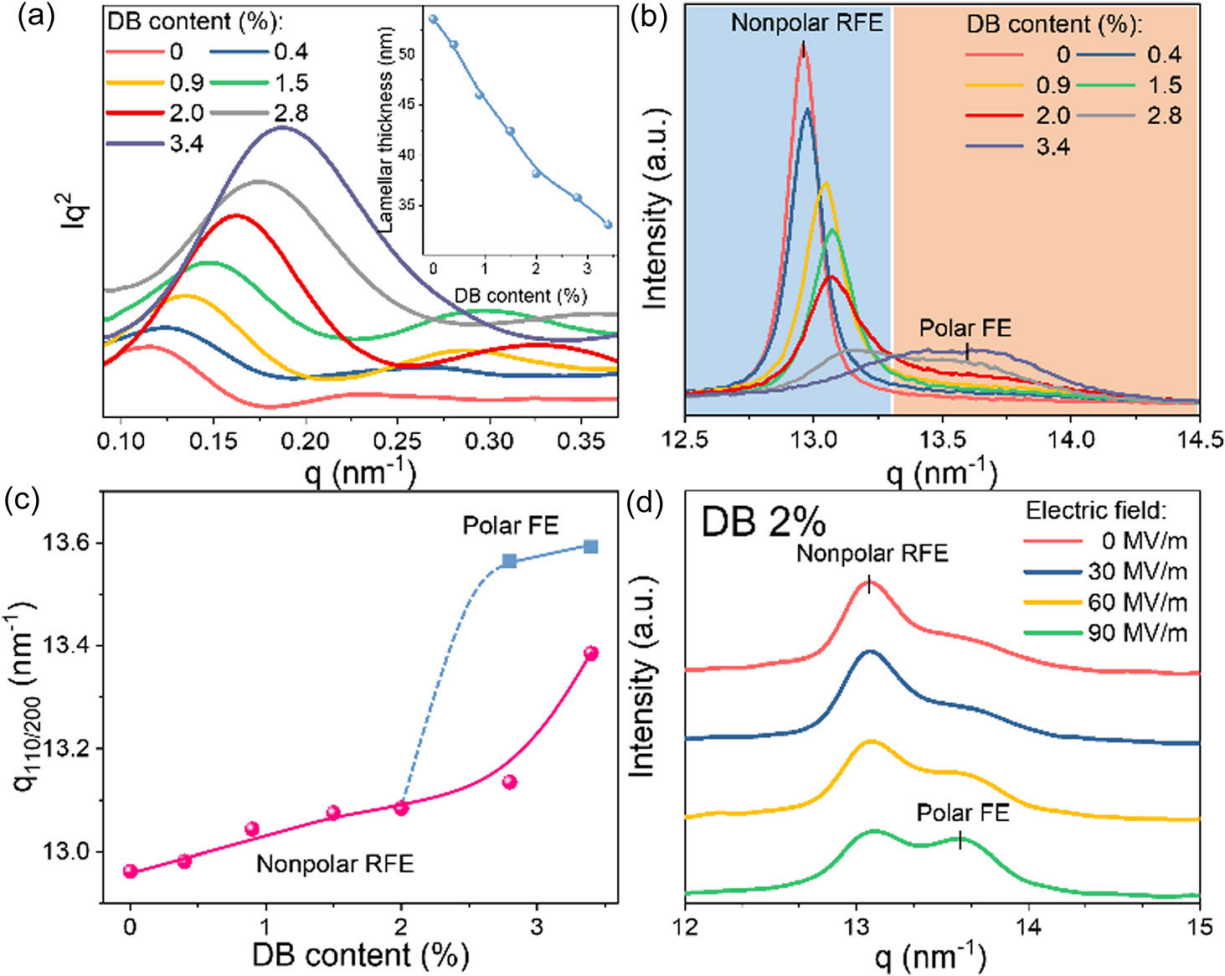
a) SAXS result of the tetrapolymers, and the inset shows the calculated lamellar spacing changes with the DB content. b) Ex situ WAXD result of the tetrapolymers and c) the summarized the d‐spacing of the (110/200) reflection changes with DB content. d) In situ XRD result for the DB 2% tetrapolymer under different electric fields.

We probed the structure changes in the tetrapolymers during the de‐HCl process. Presented in **Figure** **3**a are the Fourier‐transform infrared (FTIR) spectra following the evolution of the tetrapolymers with DB content from 0 to 3.4 mol%. The TGTG′ and T_3_GT_3_G′ peaks at 607, 866, and 1234 cm^−1^ gradually vanished with DB content (1717 cm^−1^). On the other hand, the increase in DB content also resulted in the increase of the polar *T* > 3 conformation (1283 cm^−1^). This confirms that tetrapolymers with high DB content (>2.0%) exhibit ferroelectric *P*–*E* loops (Figure 2). Based on the FTIR result, we carried out AFM‐IR mapping on the tetrapolymer with 2 mol% DB and the terpolymer at the IR frequencies of 1283 cm^−1^ (Figure 3b,c) and 1717 cm^−1^ (Figure 3d,e), corresponding to all trans conformations (*T* > 3) and DB groups, respectively. As expected, the terpolymer does not show DB IR signal (Figure 3d). On the other hand, the tetrapolymer with 2 mol% DB shows that the DB‐rich regions (Figure 3e) are also the polar phase‐rich regions (Figure 3c). From the chemical structure point of view, the planar DB unit and smaller monomer size favor the conformations with the planar zigzag polar (all trans) structures. Compared with the polar regions in the terpolymer (Figure 3b), the polar phase domains are smaller and more evenly dispersed in the tetrapolymer (Figure 3c). This is also in line with the observation in the SAXS (**Figure** **4**a) that the lamellar spacing of the tetrapolymers decreases with the DB content. All these results lead to a high polar‐entropy in the 2 mol% DB tetrapolymer.

We also performed wide‐angle X‐ray diffraction (WAXD) analyses for the tetrapolymers. Presented in Figure 4b is the ex situ WAXD curves with different DB contents at a scattering vector (*q*) range around (110/200) diffraction, indicating a gradual phase change from a nonpolar phase to a polar FE phase with DB content. At the DB content of 2 mol% and below, the *q*_(110/200)_ increase linearly with the DB content (Figure 4c), along with the peak intensity becoming weaker and the peak width becoming broader (Figure 4b). The results of reduced (110/200) interchain spacing are consistent with the FTIR data of increased all‐trans polar bonds and reduced TGTG′ and T_3_GT_3_G′ nonpolar conformations with DB content in the tetrapolymers. The broader peak at a high DB content may indicate the formation of polar domains in the crystalline phase. When the DB content reaches around 2.8%, a second high‐*q* peak appeared at a higher position (see the mark in Figure 2b), indicating the formation of the FE phase, while the low‐*q* peak became even more broad. Afterward, the newly formed high‐*q* peak moves to a higher angle, while the peak kept its broad and dispersed shape, which further confirms a polar phase with broadly distributed polar‐domain sizes after more DB units were inserted into the crystalline plane. The high ECE performance of DB 2% tetrapolymer occurs at the critical endpoint of such a nonpolar to polar phase transition.^[^ ^29^, ^30^, ^31^ ^]^

Intriguingly, such significant phase changes can also be achieved by electric poling and thus enhancing ECE performance. As shown in the in situ WAXD measurement for DB 2% tetrapolymer under different electric fields (Figure 4d), applying electrical poling at 60 MV m^−1^ induces a high‐*q* polar peak from the original low‐*q* nonpolar peak. The transformation from the nonpolar phase to polar phase at low electric fields, including dynamically pulling DB units in and out from the crystalline and crystalline‐amorphous interfaces,^[^ ^34^, ^35^ ^]^ simultaneously enhances the ECE and electroactuation in this high entropy tetrapolymer and thus endows giant cross‐energy couplings at low fields.

## 3 Conclusions

In summary, the large energy barriers between the nonpolar and polar phases in relaxor ferroelectrics pose challenges to realize the dual‐functionalities of large ECE and large electroactuation at low electric fields. By tailoring the P(VDF‐TrFE‐CFE) terpolymer compositions, we show that the tetrapolymer P(VDF‐TrFE‐CFE‐DB) formed by de‐HCl process can be transformed to a normal ferroelectric with a diffused phase transition, which exhibits a large ECE and large electroactuation at low electric fields. The tetrapolymer 67/33/4.6/2 mol% generates an EC DS of 100 J kg^−1^ K^−1^ at 100 MV m^−1^, which is on‐a‐par with the SOA EC polymer, and an in‐plane electroactuation *x*_1_ of 2.75% at 70 MV m^−1^, nearly double that of the SOA EC polymer. Ferroelectric polymers with such a high performance can provide an attractive EC heat pump design that pumps heat between the cold end and heat sink without external means.

## 4 Experimental Section

4.1

4.1.1

##### Polymerization of P(VDF‐TrFE‐CFE) Terpolymers

The terpolymers were synthesized following the procedure described in our previous patent.^[^ ^36^ ^]^ In a 3 L stirred reactor under vacuum filled with degassed and deionized water containing a hydroxypropylmethyl cellulose as dispersing agent, a mixture of TrFE and VDF was charged. Then, the reactor was heated to the desired initiation temperature, which was maintained throughout the polymerization at a value close to 44 °C. n‐Propyl peroxydicarbonate as the initiator was then injected into the reactor and the reaction began. The consumption of the monomers led to a decrease in pressure that was compensated by continuously feeding the reactor with a VDF/TrFE/CTFE mixture. The pressure was thus maintained in the range of 80 to 110 bar. When the targeted quantity of the feed mixture was reached, the injection was stopped, and the reactor was cooled down and degassed. The product was unloaded and recovered as a slurry. This slurry was filtered, and the wet powder was washed several times in pure DI water. Finally, the powder was dried in an oven at 60 °C until constant weight.

##### Preparation of P(VDF‐TrFE‐CFE‐DB) Tetrapolymer Films

P(VDF‐TrFE‐CFE‐DB) tetrapolymers were synthesized using the postfunctionalization process. We used the tertiary amine, triethylamine (TEA), as the weak base in the de‐HCl reaction to suppress the possible amination side reactions. Specifically, in a 20 mL vial, 0.5 g of P(VDF‐TrFE‐CFE) terpolymer was dissolved in 10 mL of *N*,*N*‐dimethylformamide (DMF, Millipore‐Sigma) at 50 °C overnight to form a homogeneous solution. Afterward, 1 mL of TEA (Millipore‐Sigma) was added to the solution to start the de‐HCl reaction. The DB content was controlled by a series of reaction times: 0, 2, 5, 7, 9, 12,15, 18, and 24 h. After the reaction, the polymer solution was immediately precipitated into 200 mL of 50/50 (vol./vol.) water–ethanol mixture to remove residual TEA and the by‐product, triethylammonium chloride salts. The solid was washed three times using a water and ethanol mixture and air blew overnight to remove the solvents. The dried polymer was redissolved in DMF and stirred overnight at room temperature. The homogeneous solution was then solution‐casted onto a clean glass substrate in a heating oven for 12 h at 60 °C. After drying, the films (about 15 μm thick) were peeled off from the substrate with the help of water tension. Afterward, the polymer films were placed in a vacuum oven and annealed at 120 °C for xxx hours.

##### Structural Characterizations

The ex situ WAXD was performed using a Malvern Panalytical Empyrean III (Cu K_α_ *λ* = 1.5418 Å). In situ WAXD measurement was carried out at the 11‐BM Complex Material Scattering Beamline of the National Synchrotron Light Source II (NSLS‐II), Brookhaven National Laboratory (BNL). The energy of the incident X‐ray beam was 17 keV. The distances between the sample and the WAXD (Pilatus 800k, Dectris, Gaden‐Dattwil, Switzerland) was 259 mm. The distance was calibrated using silver behenate with the first‐order reflection at a scattering vector *q *= (4*π*sin*θ*)/*λ* = 1.076 nm^−1^ (*θ* is the half scattering angle). FTIR spectroscopy was carried out on a Bruker VERTEX 70 with the attenuated total reflection (ATR) mode, with 256 scans and the resolution of 2 cm^−1^. Photothermal AFM‐IR measurements were carried out using a Bruker Dimension IconIR with PR‐UM‐CnIR probes (*k* = 0.2 N m^−1^, resonance frequency = 13 kHz). Samples were scanned in the contact mode, with the IR pulse repetition rate modulated at a contact resonance frequency of ≈181 kHz. Areas of 2 μm × 2 μm were scanned at a scan rate of 0.8 Hz, with images acquired at a resolution of 256 pixels per line. The DB contents with different reaction times were determined by ^1^ H and ^19^ F NMR with the solvent of acetonitrile‐d3. Figure S4, Supporting Information, showed the NMR result, indicating the content of the DB unit increased linearly within the first 15 h. After 15 h of the reaction, most of the CFE units (3.6%) were substituted into DB located on the polymer main chains. The thermal property of the polymers after the de‐HCl reaction was characterized by a TA Q2000 differential scanning calorimeter (DSC, TA Instruments) under a nitrogen flow (50 mL min^−1^) with heating and cooling rates of 10°C min^−1^.

##### Dielectric Characterization

Prior to the dielectric measurements, 50 nm‐thick gold electrode with 3 mm diameter (0.0706 cm^2^) was deposited on both sides of the film using an EMITECHK550X sputter coater. Temperature‐scanned dielectric spectroscopy was performed on a Hewlett–Packard (HP) 4284A LCR meter with the frequency range of interest. The samples were installed in a temperature environment chamber, and the measured temperature range was −100 to 100 °C with a ramping rate of 2 °C min^−1^. *P*–*E* loops were measured using a modified Sawyer‐Tower circuit at 10 Hz with a high‐voltage amplifier system (Trek 61°C), with varying the electric fields and measured at different temperatures.

##### Electrocaloric Effect Characterization

The ECE of the polymer films was characterized using a specifically designed calorimeter with a heat flux sensor.^[^ ^6^ ^]^ Prior to measurement, both surfaces of the samples are sputter‐coated round‐shaped gold electrodes with an over‐lapping area of 0.119 cm^2^. To apply voltage to the sample film, silver wires were glued on the top and bottom surfaces using conductive silver paste. The bottom surface (with electrode) is directly attached to the heat flux sensor to ensure good thermal contact. See the detailed discussion for the ECE measurement in the Supporting Information.

## Conflict of Interest

The authors declare no conflict of interest.

## Author Contributions

**Guanchun Rui**: conceptualization (lead); data curation (lead); formal analysis (lead); writing—original draft (lead). **Wenyi Zhu**: data curation (lead); formal analysis (equal); methodology (lead). **Li Li**: data curation (equal); formal analysis (equal); writing—original draft (equal). **Jongcheol Lee**: data curation (equal); formal analysis (equal); methodology (equal). **Yiwen Guo**: data curation (equal); formal analysis (supporting; methodology (equal); resources (equal). **Qin Zou**: data curation (equal); methodology (equal); validation (equal). **Siyu Wu**: data curation (equal); methodology (equal); software (equal). **Ruipeng Li**: data curation (equal); formal analysis (equal); methodology (equal). **Thierry Lannuzel**: resources (lead). **Fabrice Domingues Dos Santos**: resources (lead). **Mark A. Aubart**: project administration (equal); resources (equal). **Seong H. Kim**: data curation (equal); project administration (equal); writing—review editing (equal). **Long‐Qing Chen**: methodology (equal). **Lei Zhu**: methodology (equal); writing—review editing (equal). **Zi‐Kui Liu**: methodology (equal); writing—review editing (equal). **Q. M. Zhang**: conceptualization (lead); funding acquisition (lead); project administration (lead); writing—original draft (lead). Guanchun Rui and Wenyi Zhu contributed equally.

## Supporting information

Supplementary Material

## Data Availability

The data that support the findings of this study are available from the corresponding author upon reasonable request.
