# Supplementary material for: Dual‐Functional High‐Entropy Polymer Exhibiting Giant Cross‐Energy Couplings at Low Fields
Source: Small Sci. 2025 Feb 22;5(6):2400624. doi: 10.1002/smsc.202400624 (PMC12168593; doi:10.1002/smsc.202400624)
Supplement: Supplementary file 1 — Supplementary Material [file SMSC-5-2400624-s001.pdf]

Supporting Information

Dual-functional ferroelectric polymer exhibiting giant cross energy couplings at low fields

Guanchun Rui,[1,‡] Wenyi Zhu,[2,‡] Li Li,[3] Jongcheol Lee,[4] Yiwen Guo,[4] Qin Zou,[5] Siyu Wu,[6] Ruipeng Li,[6] Thierry Lannuzel,[7] Fabrice Domingues Dos Santos,[7] Mark A. Aubart,[1] Seong H. Kim,[4] Long-Qing Chen,[3] Lei Zhu,[5] Zi-Kui Liu,[3] and Q. M. Zhang[2,*]

[1]*Arkema Inc., 900 First Avenue, King of Prussia, PA, 19406, USA*

[2.]*School of Electrical Engineering and Computer Science, Materials Research Institute, The Pennsylvania State University, University Park, PA, 16802, USA*

[3]*Department of Materials Science and Engineering, The Pennsylvania State University, University Park, PA, 16802, USA*

[4]*Department of Chemical Engineering, The Pennsylvania State University, University Park, PA, 16802, USA*

[5]*Department of Macromolecular Science and Engineering, Case Western Reserve University, Cleveland, OH, 44106, USA*

[6]*National Synchrotron Light Source II, Brookhaven National Laboratory, Upton, NY, 11973, USA*

[7]*Arkema-Piezotech Rue Henri-Moissan, Pierre-Benite Cedex 69493, France*

*Email:qxz1@psu.edu

‡ These authors contributed equally: Guanchun Rui, Wenyi Zhu.

Detailed discussion for the electrocaloric effect (ECE) characterization

The details of the ECE isothermal entropy change (ΔS) measurement can be found in a previous report.[S1] The EC heat Q_E generated by the EC polymer film in response to the voltage on-and-off process is compared with the heat Q_h generated by a reference resistor R from the metal electrode on the surface of the polymer film. When a voltage, V, with a pulse time duration, t, applies to the resistor heater R, it produces a joule heat $Q_h=(V^2/R)t$. The heat generated is detected by a heat flux sensor directly attached to the sample film surface. Now, if the ECE film under an applied electric field also generates the same amount of heat as detected by the same flux sensor, then the heat Q_E from the ECE material is equal to Q_h. From $Q_h = Q_E = T\Delta S$, where T(Kelvin) is the environment temperature (sample temperature), the isothermal entropy changes ΔS can be obtained. In the calibration process, different values of Q_h were measured by changing voltage V on the standard resistor R, from V=1 V to 5 V. Thus $Q_h=(V^2/R)t$ over the whole EC measurement range is calibrated, see Figure S1, the calibration coefficient is a constant (0.040 to 0.042 J/V^2) in the whole Q_h range calibrated. That is, the calibration coefficient measured at one voltage V can be used for the whole Q_E range in the EC ΔS measurement.

In this study, the EC polymer film thickness is about 15 um. From the thermal conductivity and the specific heat of the EC polymer, it can be estimated that such polymer films can reach thermal equilibrium within 0.01 seconds (between top and bottom surfaces), see Eq. S1,

$$\delta = \sqrt{\frac{2\alpha}{\omega}} \qquad \text{(S1)}$$

$$\text{and } \omega = \frac{2\alpha}{\delta^2} = 2\pi f \qquad \text{(S2)}$$

where δ is the thermal diffuse length, for our cases, the film thickness is 15 μm, and $\alpha = \frac{k}{\rho C_p}$, where k is the thermal conductivity, ρ is the density and Cp is the heat capacity of the polymer films. From Eq. S2, it can be deduced the frequency f=105 Hz for δ=15 μm, which corresponding to the thermal equilibrium time < 0.01s. Thus, for the EC ΔS measurement, the EC polymer films were in thermal equilibrium across the film thickness.

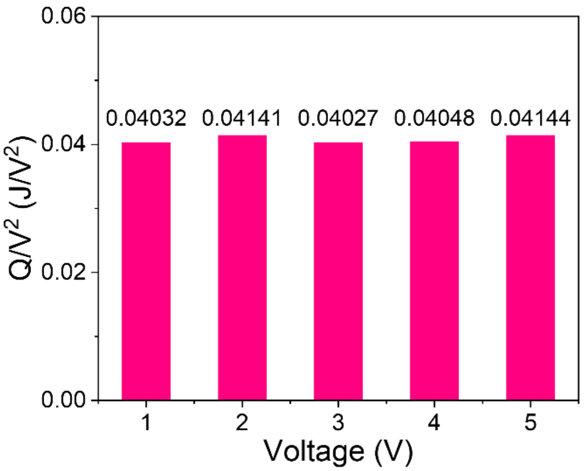

Figure S1. The calibration coefficient (Q/V^2) of the heat signals measured by the flux sensor and applied voltage V (at different voltages).

As shown in Ref. S2, ΔT and ΔS are related through the specific heat $C_{BC}(E_h, T)$ which is the specific heat at E_h along the path BC in Figure S2,[S2]

$$\Delta S = -\int_{T1}^{T2} [C_{BC}(E_h, T)/T]\, dT \qquad (S3)$$

where $\Delta T = T_2 - T_1$. Because we do not have an instrument which can measure $C_{BC}(E_h, T)$, the specific heat under electric field E_h, and also specific heat of the films in the study changes with temperature, we can't deduce ΔT deduced from Eq. (S3).

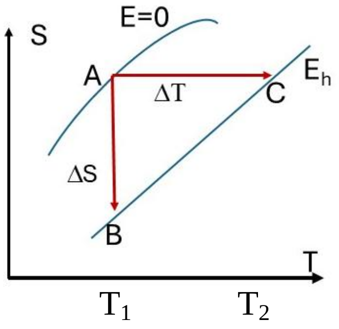

Figure S2. Schematic S-T diagram for the electrocaloric effect of ΔS and ΔT between E=0 and $E=E_h$.

On the other hand, if $C_{BC}(E_h, T)$ does not show much change with temperature in the temperature range of ΔT and $\Delta T << T$, Eq. S(3) is reduced to

$$\Delta S = - C_{BC}(E_h, T)\, \Delta T/T \qquad (S4)$$

In the literature such as using Maxwell equation to deduce the ECE, $C_{BC}(0, T)$,[S3] which is the specific heat measured at E=0, is often used to relate ΔS and ΔT. In the studies of EC polymers, we also used $C_{BC}(0, T)$ to estimate ΔT from ΔS measured from the calorimeter described here. ΔT of those EC polymer films have also been measured using thermocouple and IR cameras.[S4-S6] In the ΔT range overlapped, within the experimental error, the results deduced from ΔS and measured using thermocouple and IR carema are the same.

Using the specific heat at E=0 and ΔS measured here, we deduce ΔT = 20 K under 100 MV/m for the 67/33/4.6/2 tetrapolymer.

Electrocaloric effect in P(VDF-TrFE-CFE-DB) 67.3/32.7/5.5-x/x tetrapolymers

We also synthesized and characterized terpolymer with even lower CFE contents such as 67.3/32.7/5.5. The dielectric properties vs temperature measured at different frequencies, Figure S3(d). this terpolymer already exhibited a broad dielectric peak at 46 ^{o}C, which position does not change with frequencies. This is very different from the terpolymer 67/33/6.6. The ECE of the tetrapolymers derived did not show much change with the DB content, see Figure S9(a), and is lower than that P(VDF-TrFE-CFE-DB) 67/33/4.6/2. We have also characterized thermal properties, see Figure S9(b). The DSC data show a transition peak at ca. 46 ^{o}C even for the 67.3/32.7/5.5 terpolymer, in addition to the tetrapolymers, likely caused by low content of CFE in the terpolymer. The tetrapolymers derived from this terpolymer composition exhibit strong

normal ferroelectric behavior and do not generate high ECE. We did not carry out other studies of the tetrapolymers here.

Figures:

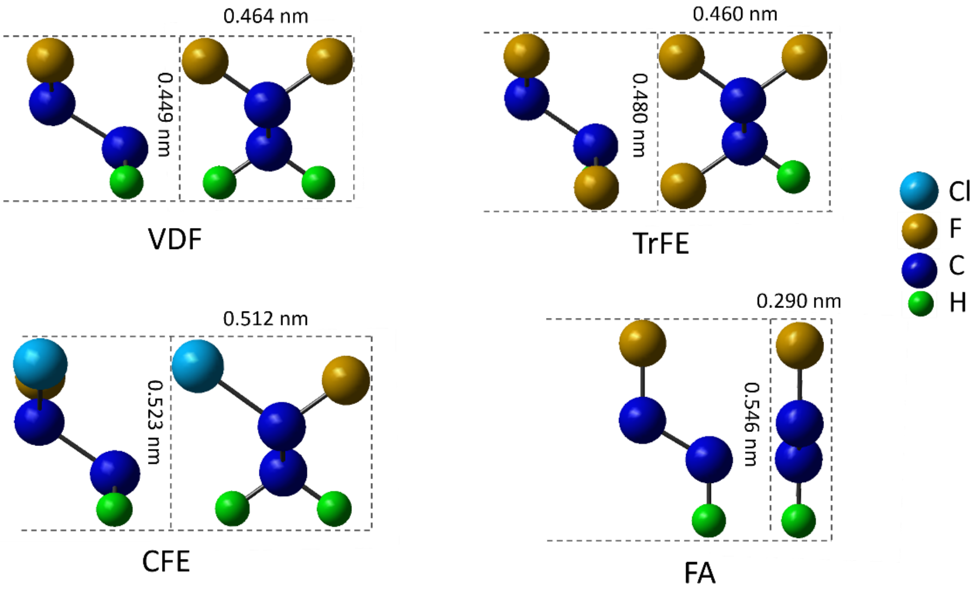

Figure S3. Schematics of the VDF, TrFE, CFE, and DB monomers and their sizes.

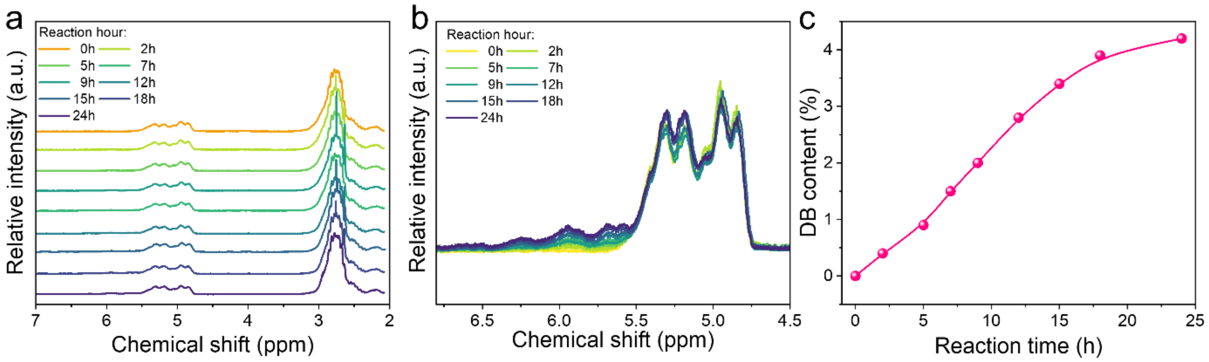

Figure S4. DB content with the increase of reaction time. The DB content was determined by ^{1}H NMR.

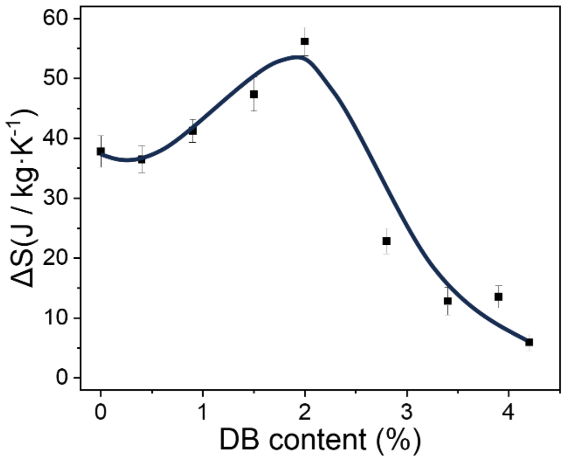

Figure S5. ΔS vs. DB content n of P(VDF-TrFE-CFE-DB) 67/33/6.6-n/n tetrapolymers under 80 MV/m, the error bar showing the mean value ± SD, n=6.

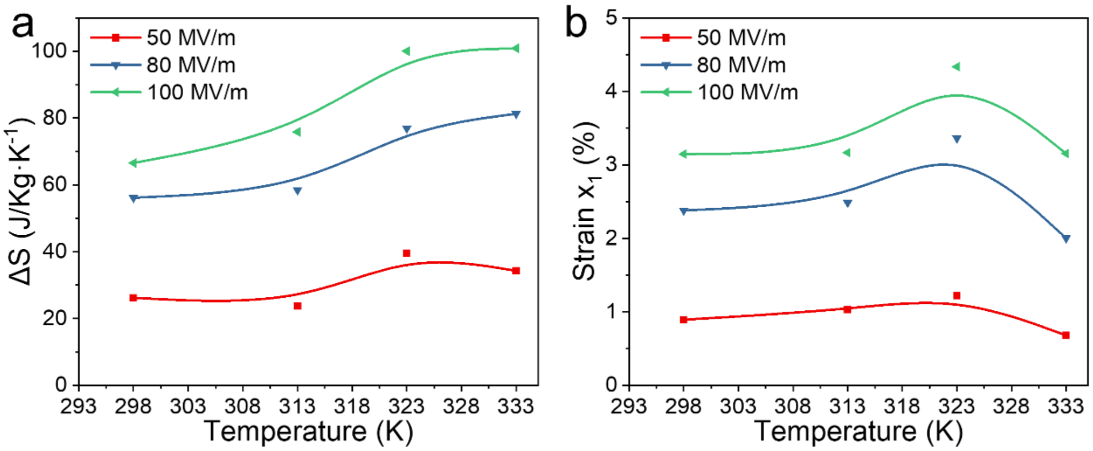

Figure S6. Electrocaloric (a) ΔS and (b) strain x_1 under different electric fields vs. temperature for the P(VDF-TrFE-CFE-DB) 67/33/4.6/2.0 mol% tetrapolymer.

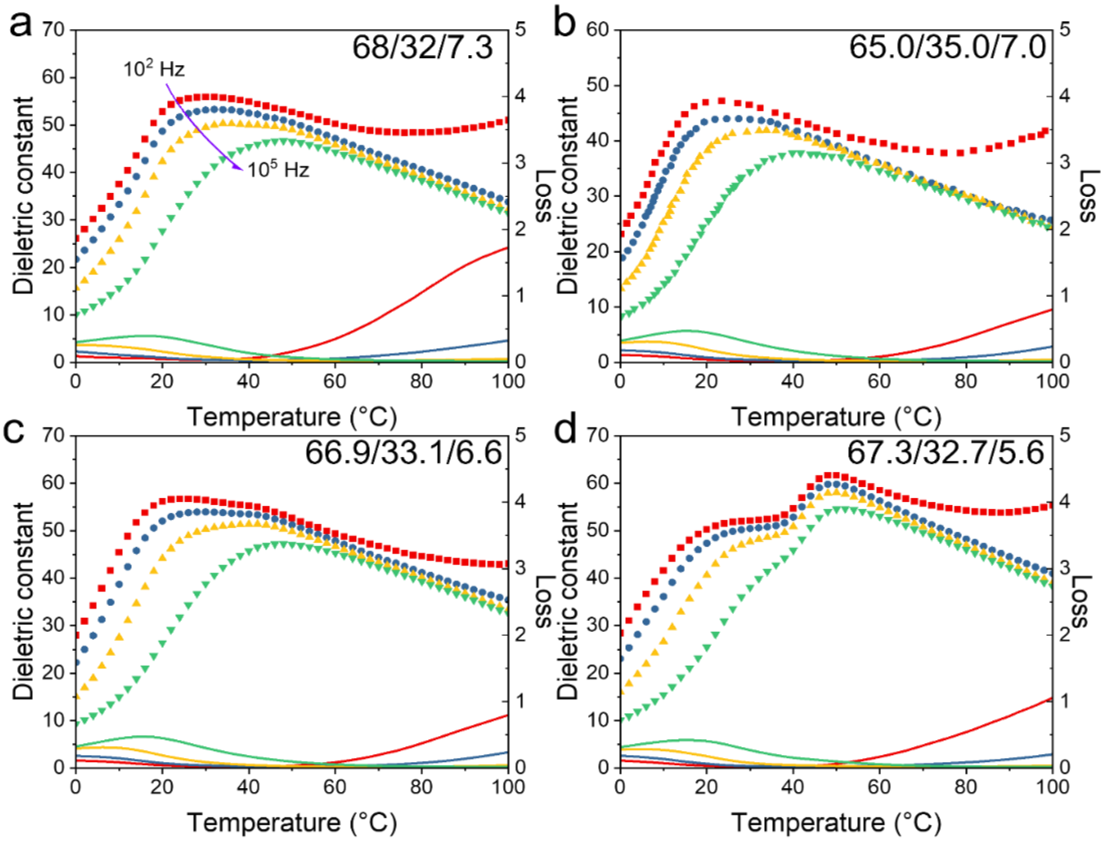

Figure S7. Dielectric properties at different frequencies vs. temperature for P(VDF-TrFE-CFE) terpolymer at compositions (a) 68/32/7.3 mol%, (b) 65/35/7 mol%, (c) 66.9/33.1/6.6 mol%, (d) 67.3/32.7/5.6 mol%.

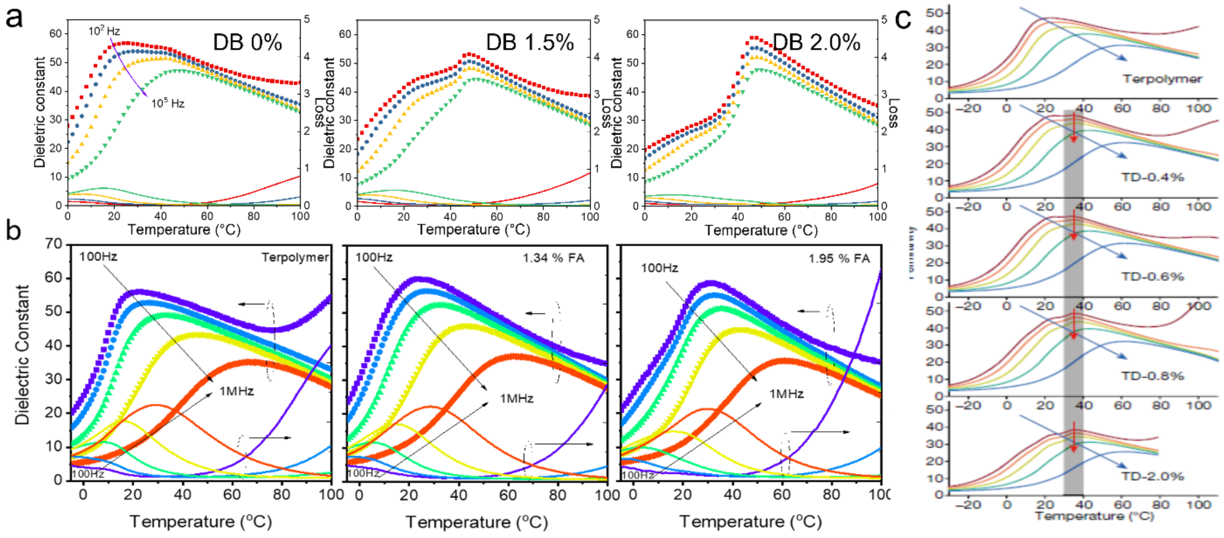

Fig. S8. The dielectric constant at different frequencies vs. temperature for the terpolymer and DB tetrapolymers of a) this study, 67/33/6.6 mol%; b) Ref. 27, Reproduced with permission.[27]

Copyright 2023, American Chemical Society, and c) Ref. 9 in the main text. Reproduced with permission.[9] Copyright 2021, Springer Nature.

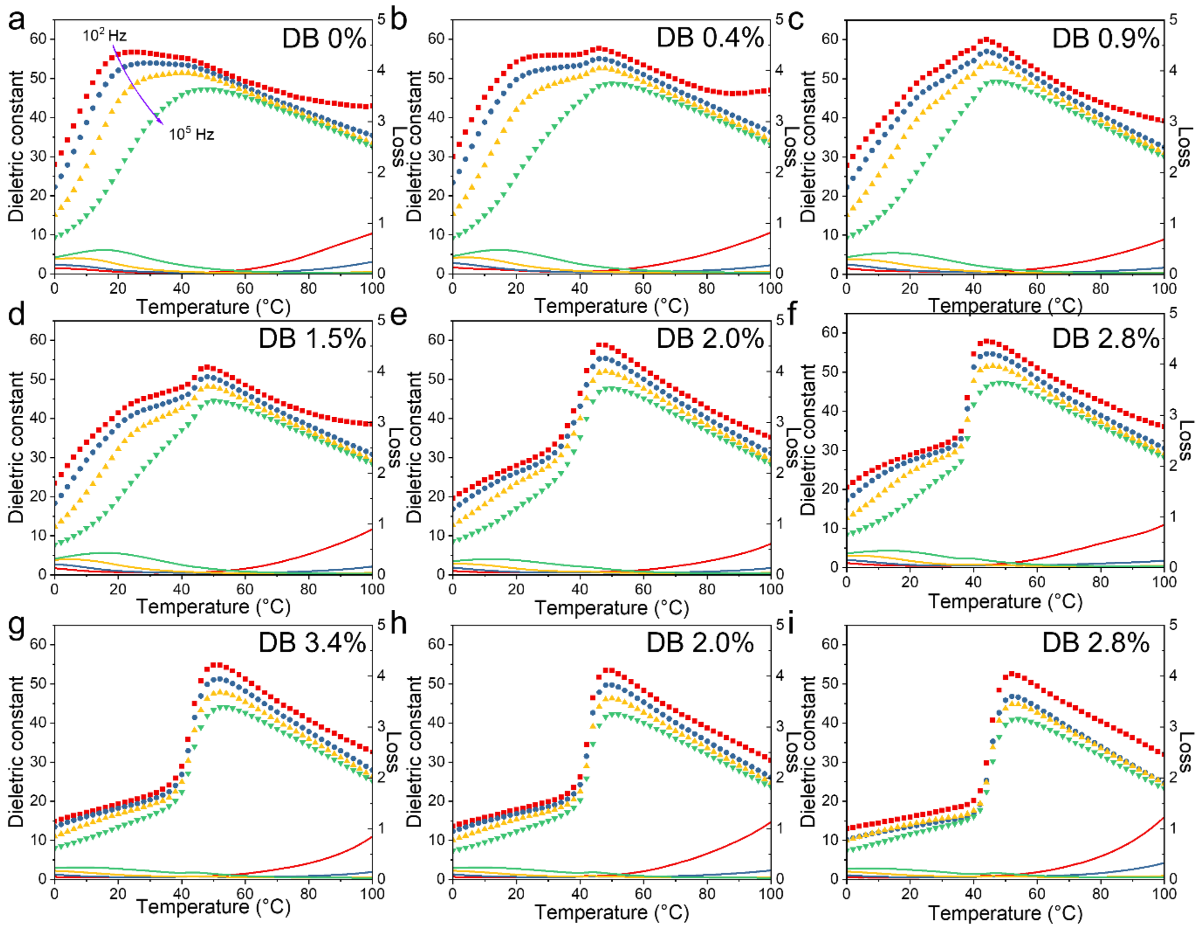

Figure S9. Dielectric properties at different frequencies vs. temperature for the tetrapolymer P(VDF-TrFE-CFE-DB) 66.9/33.1/6.6-x/x with different DB contents.

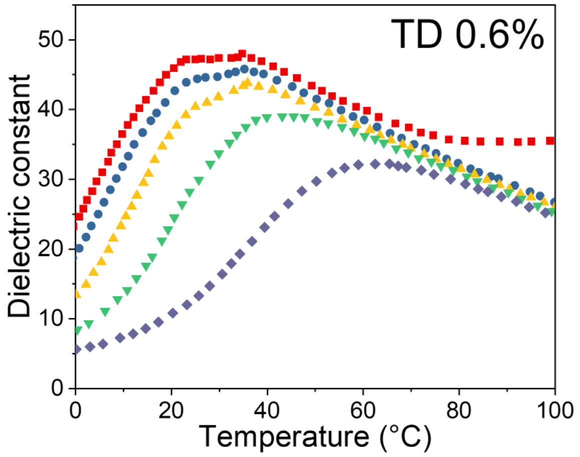

Figure S10. The dielectric constant at different frequencies vs. temperature of the tetrapolymer, which shows the highest ECE in the main text, Ref. 9.

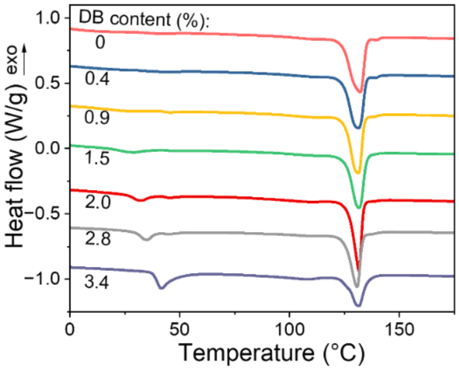

Figure S11. DSC measurements of P(VDF-TrFE-CFE-DB) 67/33/6.6-n/n tetrapolymers during the heating rate of 10 K/min.

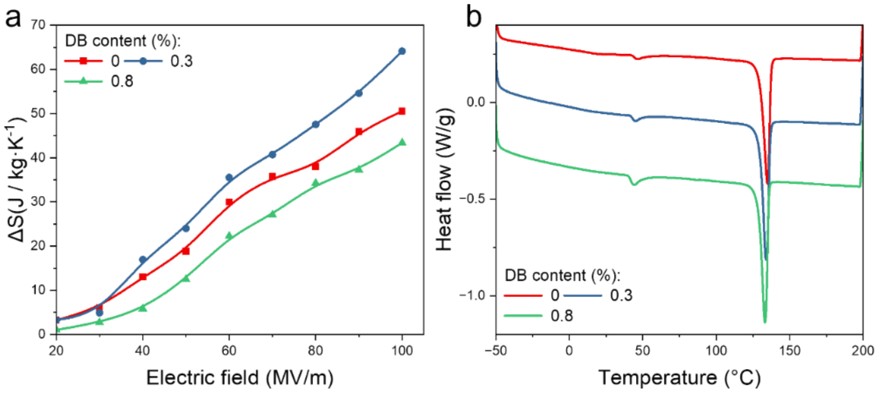

Figure S12. (a) The EC ΔS of the 67.3/32.7/5.6 terpolymer and 67.3/32.7/5.6-n/n tetrapolymers (where n = 0.3 and 0.8 mol%), and (b) DSC of the 67.3/32.7/5.6 terpolymer and 67.3/32.7/5.6-n/n tetrapolymers (where n = 0.3 and 0.8 mol%).

Reference

S1. Li X, Qian X-S, Lu S, Cheng J, Fang Z, Zhang QM. (2011) Tunable temperature dependence of electrocaloric effect in ferroelectric relaxor poly(vinylidene fluoride-trifluoroethylene-chlorofluoroethylene terpolymer. *Appl. Phys. Lett.* **99**(5)**:** 052907.

S2. Pirc R, Kutnjak Z, Blinc R, Zhang Q.M.(2011) Upper bounds on the electrocaloric effect in polar solids. *Appl. Phys. Lett.* **98**(2)**:** 021909.

S3. Chen X, Shvartsman V V, Lupascu D C, Zhang Q. M. (2022) Perspective: Electrocaloric cooling – from materials to devices. *J. Appl. Phys*. **132:** 240901.

S4. Guo D, Gao J; Yu Y-J; Santhanam S; Fedder V; McGaughey A J H; Yao S. C. (2014) Electrocaloric characterization of a P(VDF-TrFE-CFE) terpolymer by infrared imaging. *Appl. Phys. Lett.* **105**: 031906.

S5. Lu S G, Rožič B, Zhang Q. M., Kutnjak Z, Pirc R, Lin M, Li X, Gorny L. (2010) Comparison of Directly and Indirectly Measured Electrocaloric Effect in the Relaxor Ferroelectric Polymers. *Appl. Phys. Lett*. **97**: 202901.

S6. Lu S G, Rožič B, Zhang Q. M., Kutnjak Z, Neese B (2011) Enhanced Electrocaloric Effect in Ferroelectric P(VDF-TrFE) 55/45 mol% Copolymer at Ferroelectric-Paraelectric Transition. *Appl. Phys. Lett.* **98**: 122906.
